# Exosomes derived from microRNA-199a-overexpressing mesenchymal stem cells inhibit glioma progression by down-regulating AGAP2

**DOI:** 10.18632/aging.102092

**Published:** 2019-08-05

**Authors:** Lei Yu, Si Gui, Yawei Liu, Xiaoyu Qiu, Guozhong Zhang, Xi’an Zhang, Jun Pan, Jun Fan, Songtao Qi, Binghui Qiu

**Affiliations:** 1 Department of Neurosurgery, Nanfang Hospital, Southern Medical University, Guangzhou 510515, P. R. China; 2 Department of Radiology, Affiliated Cancer Hospital and Institute of Guangzhou Medical University, Guangzhou 510095, P. R. China

**Keywords:** glioma, microRNA-199a, AGAP2, mesenchymal stem cells, exosomes, chemosensitivity, aging, gerontology, age-related-diseases

## Abstract

Accumulating evidence has implied that microRNAs (miRNAs) are implicated in glioma progression, and genetically engineered mesenchymal stem cells can help to inhibit tumor growth of glioma. Herein we hypothesized that miR-199a could be delivered by mesenchymal stem cells to glioma cells through exosomes and thus prevent the glioma development by down-regulating ArfGAP with GTPase domain, ankyrin repeat and PH domain 2 (AGAP2). The expression pattern of miR-199a and AGAP2 was characterized in glioma tissues and cells using RNA polymerase chain reaction quantification, immunohistochemical staining and Western blot assays. Mesenchymal stem cells transfected with miR-199a mimic or their derived exosomes were co-cultured with U251 cells. The biological behaviors as well as chemosensitivity of U251 cells were assessed to explore the involvement of miR-199a/AGAP2 in glioma. MiR-199a was poorly expressed in glioma tissue and cells while AGAP2 was highly expressed. Mesenchymal stem cells delivered miR-199a to the glioma cells *via* the exosomes, which resulted in the suppression of the proliferation, invasion and migration of glioma cells. Besides, mesenchymal stem cells over-expressing miR-199a enhanced the chemosensitivity to temozolomide and inhibited the tumor growth *in vivo*. Taken together, mesenchymal stem cell-derived exosomal miR-199a can inhibit the progression of glioma by down-regulating AGAP2.

## INTRODUCTION

Accompanied with a high mortality rate, gliomas are a type of brain and spinal cord tumor that originate from glia cells and can develop into malignant neoplasms [1, 2]. Surgical resection, radiation therapy and chemotherapy are important methods for the management of patients with gliomas [3]. Due to the tendency of glioma cells to infiltrate the brain and migrate long distances from the tumor, it is impossible to rely on surgical resection to completely remove the tumour [4]. Furthermore, in recurrent high-grade gliomas, there are very few satisfactory treatment methods with a desirable prognosis [5]. Recently, increasing evidence has indicated the beneficial application of mesenchymal stem cells (MSCs) as a potential approach for treating gliomas [6, 7]. MSCs can produce and secrete exosomes that can be useful and conducive in glioma treatment [8, 9]. Therefore, it is promising to further explore a novel treatment of glioma based on MSCs-derived exosomes.

ADP-ribosylation factors GTPase-activating proteins (Arf GAPs) have the catalytic function of hydrolyzing GTP bound to Arf, accompanied by multiple cellular functions [10]. Arf GAPs play a role in forming membrane vesicles that traffic substances between subcellular membranous organelles [11]. Arf GTPase-activating protein-2 (AGAP2) is able to mediate endosomal trafficking and is found to be over-expressed in many human cancers [12]. Interestingly, based on the bioinformatics prediction in our study, AGAP2 is found to be a target gene of microRNA-199a (miR-199a). This finding thereby led to our hypothesis regarding the potential association of miR-199a and AGAP2 with glioma progression via interaction. MiRNAs are short, non-coding RNAs that have the ability to mediate the expression of their target genes. It has been demonstrated that abnormal aberrant expression of miRNAs is commonly discovered in human tumorigenesis [13]. Accumulating evidence has revealed that miR-199a is a cancer-related miRNA, and its over-expression could impede cancer progression in renal cancer and papillary thyroid carcinoma [14, 15]. Notably, exosomes, which serve as natural vehicles of intercellular communication, are capable of delivering proteins, mRNAs, as well as miRNAs, in order to modulate various physiological and pathological processes [16]. Based on the previous reports and findings, we thereby hypothesized that MSCs-derived exosomes could deliver miR-199a to glioma cells and thus prevent the tumorigenic progression of glioma *via* the down-regulation of AGAP2. Thus, this study was aimed at investigating the underlying mechanism of MSCs-derived exosomes delivered miR-199a in glioma, with the involvement of AGAP2.

## RESULTS

### miR-199a may regulate AGAP2 gene in glioma

**Figure 1:**
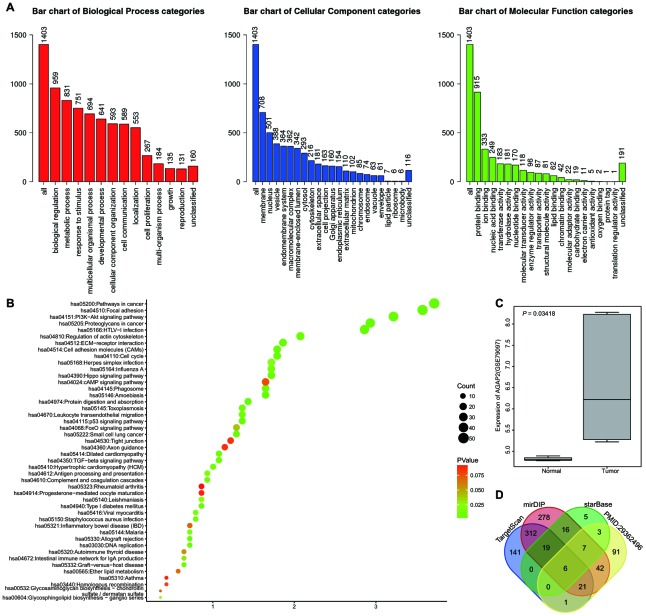
**miR-199a may regulate the AGAP2 gene in gliomas.** (**A**) GO enrichment analysis on the differential genes in glioma-related profiles. The abscissa represents GO items and the ordinate represents the number of the differential genes. (**B**) KEGG functional enrichment analysis of the DEGs in glioma expression profile. The abscissa represents GeneRatio and the ordinate represents the KEGG items. The circle color and circle size indicate the p value and Count value, respectively. (**C**) the expression of AGAP2 gene in GSE79097 profile. The abscissa indicates the sample type and the ordinate indicates the gene expression. (**D**) the prediction of regulatory miRNAs of AGAP2. The four ellipses in the figure represent the prediction results from TargetScan database, mirDIP database and starBase database, and the expression of miRNAs in exosomes reported in the relevant literature. The middle part represents the intersection of four sets of data.

The glioma-related expression dataset of GSE79097 was retrieved from the GEO database. A large number of differentially expressed genes (DEGs) were obtained to analyze the gene expression differences between glioma samples and normal samples. GO functional enrichment analysis revealed the main enrichment of the DEGs in "biological regulation", "membrane" and "protein binding" items (Figure 1A). Further enrichment analysis of KEGG items revealed that these DEGs were mainly concentrated in the signaling pathways of "Pathways in cancer", "Focal adhesion" and "PI3K-Akt signaling pathway" (Figure 1B). These results indicate that the DEGs are likely to be implicated in glioma development. Among these DEGs, it was noted that AGAP2 was highly expressed in gliomas (Figure 1C). Current studies have revealed that AGAP2 is involved in multiple tumor disease regulation including gliomas [17–19]. In order to further understand the upstream regulation mechanism of AGAP2 gene in glioma, Target Scan and other databases were applied to predict the upstream regulatory miRNAs of AGAP2. Meanwhile, the miRNAs expressed in the exosomes of human mesenchymal stem cells (hMSCs) were retrieved in a previously published literature [20]. The predicted results of the databases were intersected with reported miRNAs in the literature (Figure 1D). Finally, six potential regulatory miRNAs of AGAP2 were obtained. Among the six miRNAs, we found that miR-199a was in highest abundance in the exosomes among the reported miRNAs. These results suggested that miR-199a is likely to target the AGAP2 gene in glioma *via* exosomes, which could ultimately prevent glioma development.

### MiR-199a is poorly expressed while AGAP2 is highly expressed in glioma tissues and cell lines

**Figure 2:**
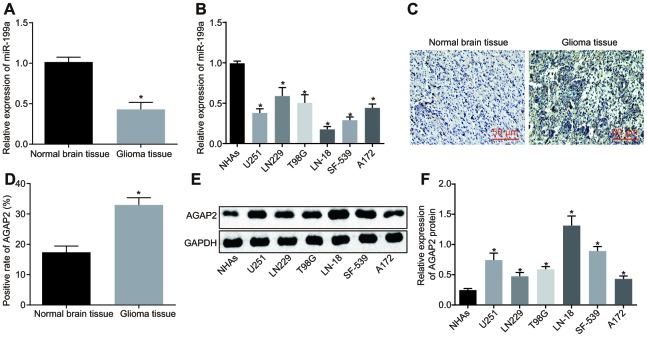
**MiR-199a is poorly expressed, while AGAP2 highly expressed in glioma tissues and cell lines.** (**A**) the expression of miR-199a in normal brain tissues and glioma tissues, determined by RT-qPCR. (**B**) the expression of miR-199a in NHAs and the glioma cell lines, determined by RT-qPCR. (**C**–**D**) the expression of AGAP2 in normal brain tissues and glioma tissues, detected by immunohistochemistry (200 ×). (**E**–**F**) the protein expression of AGAP2 in NHAs and different glioma cell lines, detected by Western blot analysis. * *p* < 0.05 compared with normal brain tissues or NHAs. The results were measurement data, presented as mean ± standard deviation. Independent sample *t*-test was used for the analysis in A and (**D**) One-way analysis of variance was used for other analyses. In tissues experiment, there were 75 glioma tissues and 17 normal brain tissues. The cell experiment was repeated three times.

The miR-199a expression in normal brain tissues, NHAs, glioma tissues and different cell lines was determined by reverse transcription quantitative polymerase chain reaction (RT-qPCR). The results displayed that miR-199a expression was lower in glioma tissues relative to that in normal brain tissues (Figure 2A), and was also significantly down-regulated in glioma cell lines compared to that in NHAs (Figure 2B). The positive expression of AGAP2 in normal brain tissues and glioma tissues were tested using immunohistochemistry. Results showed that AGAP2 was represented as a tan coloration in the cytoplasm and cell membrane and was highly expressed in glioma tissues (Figure 2C). Compared with the normal brain tissues, the positive expression rate of AGAP2 in glioma tissues was significantly higher (Figure 2D). Moreover, the protein expression of AGAP2 was detected by Western blot analysis in NHAs and cancer cell lines. The results revealed that the AGAP2 protein expression was higher in the glioma cell lines than that in the NHAs (Figure 2E, 2F).

### Over-expression of miR-199a inhibits the migrative, invasive and proliferative abilities of U251 cells

**Figure 3:**
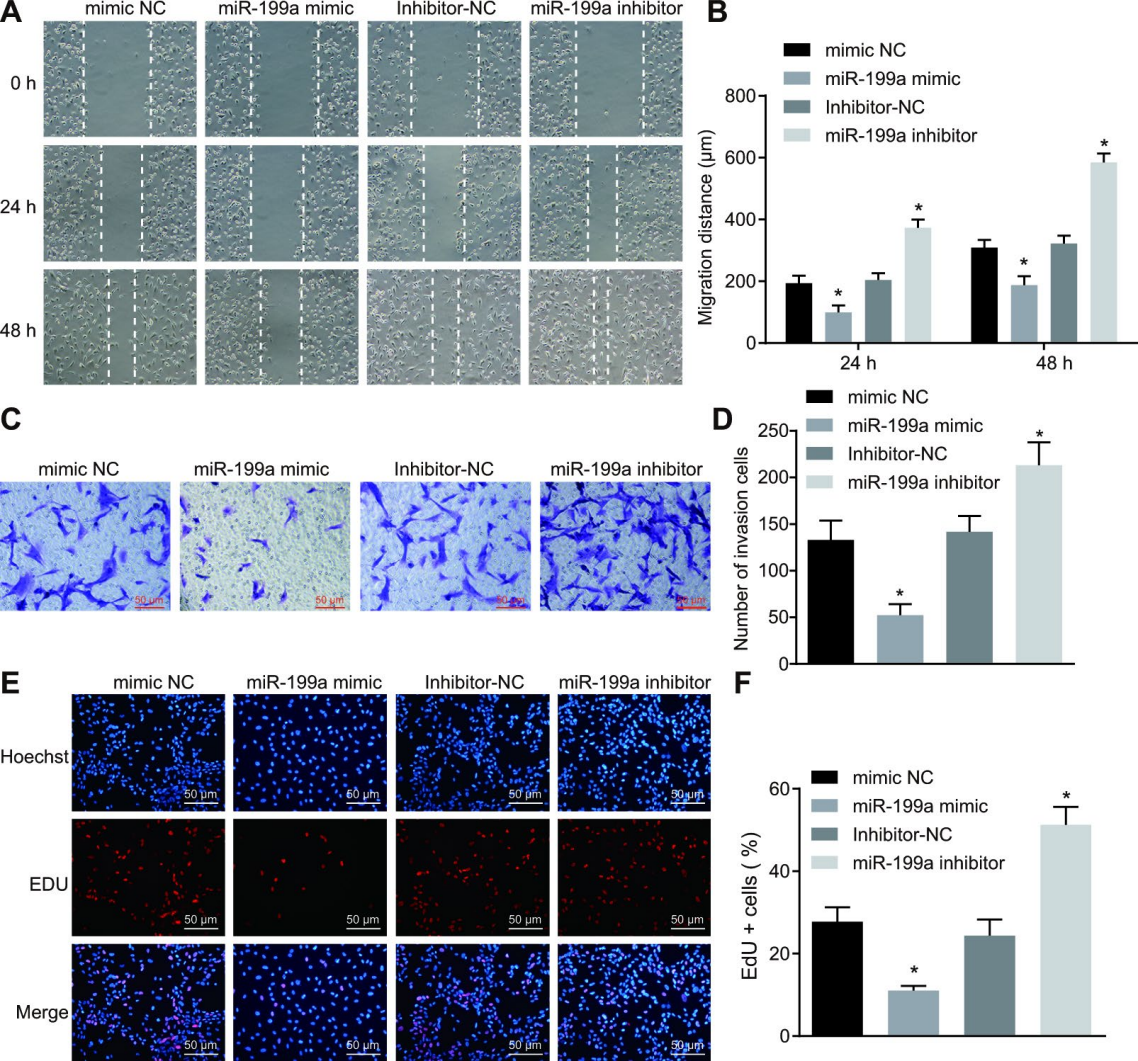
**Over-expression of miR-199a inhibits the migration, invasion and proliferation of U251 cells.** (**A**–**B**) the migration ability of U251 cells measured by scratch test (Scale bar = 100 μM). (**C**–**D**) the invasion ability of U251 cells evaluated by Transwell assay (Scale bar = 50 μM). (**E**–**F**) the proliferation ability of U251 cells assessed by EdU assay (Scale bar = 50 μM). * *p* < 0.05 compared with the corresponding NC. The results were measurement data, presented as mean ± standard deviation. Comparison between two groups using independent sample *t* test. The experiment was repeated three times. mimic-NC, cells transfected with mimic-negative control; miR-199a mimic, cells transfected with miR-199a mimic; inhibitor-NC, cells transfected with inhibitor-negative control; miR-199a inhibitor, cells transfected with miR-199a inhibitor.

The functions of miR-199a over-expression on the proliferative, migrative as well as invasive abilities of glioma cells were analyzed by 5-ethynyl-2'-deoxyuridine (EdU) assay, scratch test and Transwell assay. The proliferative, invasive and migrative abilities of U251 cells decreased significantly when transfected with miR-199a mimic. However, we found a significant upregulation, when miR-199a was inhibited in U251 cells (Figure 3). Therefore, we demonstrated that overexpression of miR-199a inhibited the migration, invasion and proliferation of U251 cells, and thus plays a crucial anti-tumor role.

### MiR-199a targets and down-regulates AGAP2

**Figure 4:**
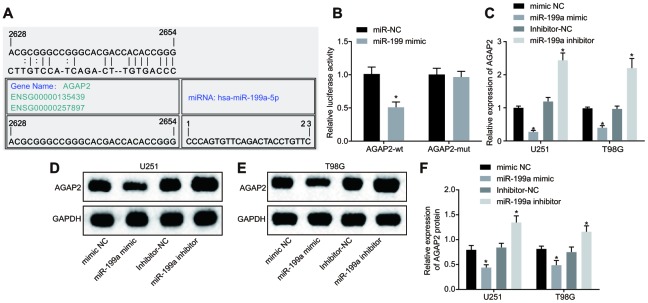
**MiR-199a directly targets AGAP2 and down-regulates its expression.** (**A**) the target binding site between miR-199a and AGAP2 was predicted through the bioinformatics website. (**B**) the results of the dual luciferase reporter gene assay. (**C**) the mRNA expression of AGAP2 in U251 and T98G cell lines with over-expressed or inhibited miR-199a, determined by RT-qPCR. (**D**–**E**) the protein expression of AGAP2 in U251 and T98G cell lines with over-expressed miR-199a, detected by Western blot analysis. (**F**) the protein expression of AGAP2 in U251 and T98G cell lines in the presence of the inhibition of miR-199a, detected by Western blot analysis. * *p* < 0.05 compared with the corresponding NC. The results were measurement data, presented as mean ± standard deviation. Paired *t*-test was used for comparison between two groups. The experiment was repeated three times. mimic-NC, cells transfected with mimic-negative control; miR-199a mimic, cells transfected with miR-199a mimic; inhibitor-NC, cells transfected with inhibitor-negative control; miR-199a inhibitor, cells transfected with miR-199a inhibitor.

The targeted binding site of AGAP2 and miR-199a was predicted through bioinformatics database (microRNA.Org; http://www.microrna.org/micrornahome.dog/) (Figure 4A). To verify that AGAP2 is the direct target gene of miR-199a, a dual luciferase reporter gene assay was carried out to test this relationship. The results showed that compared with the control group, the luciferase activity of' AGAP2 wild-type (WT) 3’-untranslated region (UTR) was significantly inhibited when treated with miR-199a (*p* < 0.05), while that of mutant (MUT) 3’-UTR was not inhibited (Figure 4B). To further verify the effect of miR-199a on AGAP2, the expression of AGAP2 in U251 and T98G cell lines after over-expression or inhibition of miR-199a was determined by RT-qPCR and Western blot analysis. In glioma cells over-expressing miR-199a, the mRNA and protein expression of AGAP2 decreased significantly (p < 0.05). However, when we inhibited miR-199a, we observed an upregulation of AGAP2 expression (Figure 4C–4F). In summary, AGAP2 was a direct target gene of miR-199a, and miR-199a down-regulated the expression of AGAP2.

### The inhibition role of miR-199a in U251 cells is mediated by down-regulation of AGAP2

**Figure 5:**
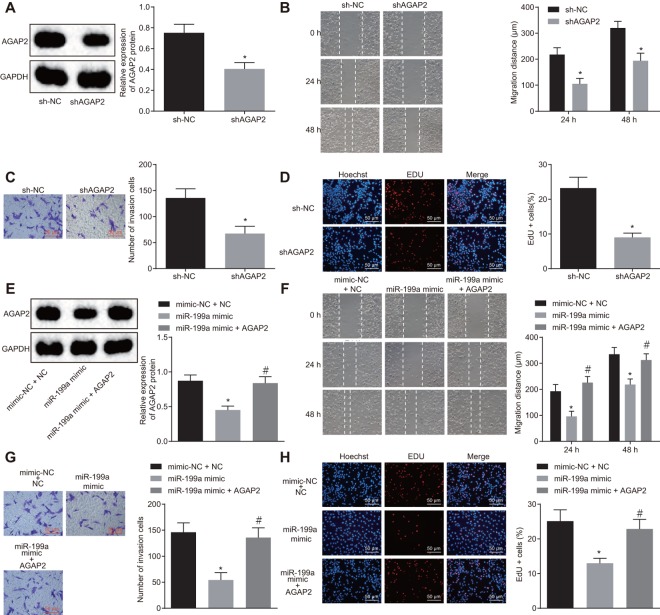
**The inhibitory role of miR-199a in U251 cells is mediated by the down-regulation of AGAP2.** (**A**) the protein expression of AGAP2 after AGAP2 silencing, detected by Western blot analysis. (**B**) the migration ability of U251 cells after AGAP2 silencing, measured by scratch test (Scale bar = 100 μM). (**C**) the invasion ability of U251 cells after AGAP2 silencing, assessed by Transwell assay (Scale bar = 50 μM). (**D**) the proliferation ability of U251 cells after AGAP2 silencing, evaluated by EdU assay (Scale bar = 50 μM); (**E**) the protein expression of AGAP2 in the presence of miR-199a mimic or sh-AGAP2 or sh-NC, detected by Western blot analysis. (**F**) the migration ability of U251 cells (Scale bar = 100 μM), measured by scratch test. (**G**) the invasion ability of U251 cells (Scale bar = 50 μM), assessed by scratch test. (**H**) the proliferation ability of U251 cells, evaluated by EdU assay. (**A**–**D**) * *p* < 0.05 compared with the sh-NC group; (**E**–**H**) * *p* < 0.05 compared with mimic-NC + sh-NC group. # *p* < 0.05 compared with the miR-119a mimic group. The results were measurement data, presented as mean ± standard deviation. Paired *t*-test was used for the analysis in A, C and D. One-way analysis of variance was used for other analyses. The experiment was repeated three times. sh-NC, cells transfected with sh-negative control; sh-AGAP2, cells transfected with sh-AGAP2; mimic-NC + sh-NC, cells transfected with mimic-negative control and sh-negative control; miR-199a mimic, cells transfected with miR-199a mimic; miR-199a mimic + sh-AGAP2, cells transfected with miR-199a mimic and sh-AGAP2.

The effect of AGAP2 on the development of glioma cells was verified by AGAP2 gene silencing. The silencing efficiency of shAGAP2 was evaluated by Western blot analysis, which revealed a significant decline of AGAP2 in U251 cells (Figure 5A). The findings by Transwell assay, scratch test and EdU assay revealed that after AGAP2 silencing, the abilities of invasion, migration and proliferation of U251 cells decreased distinctly (Figure 5B–5D). Moreover, the co-transfection of miR-199a mimic and over-expressed AGAP2 was performed in glioma cells. It was shown that over-expression of miR-199a alone could inhibit the expression of AGAP2, and significantly attenuate the proliferation, migration and invasion of glioma cells (Figure 5E–5H). However, when AGAP2 was over-expressed in the cells transfected with the miR-199a mimic, we found the opposite effect of miR-199a on the glioma cells (Figure 5F–5H). These results suggested that the inhibitory role of miR-199a in U251 cells was mediated by down-regulation of AGAP2.

### Exosomes are successfully extracted from hMSCs

**Figure 6:**
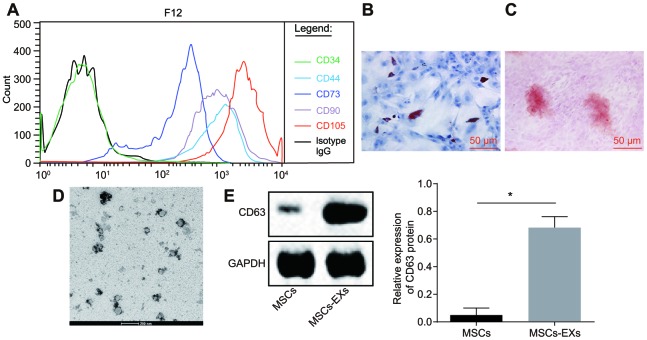
**Exosomes are successfully isolated from the supernatant of hMSCs.** (**A**) the characterization of surface antigens of hMSCs, detected by flow cytometry. (**B**–**C**) the adipogenic differentiation of hMSCs stained with Oil Red O. (**C**) the osteogenic differentiation of hMSCs stained with Alizarin red S. Green, arrows indicate positive cells (Scale bar = 50 μM). (**D**) the characterization of exosome structure under the electron microscope (Scar bar = 200 nm). (**E**) the identification of the exosome surface marker CD63 by Western blot assay. * *p* < 0.05. The results were measurement data, presented as mean ± standard deviation. Paired *t*-test was used for the analysis in E. The experiment was repeated three times.

The hMSCs of the third generation were isolated and cultured. FITC-labeled mouse anti-human antibodies against CD34, CD44, CD73, CD90 and CD105 were used to identify surface antigens by flow cytometry. CD44, CD73, CD90 and CD105 were used as markers of hMSCs and CD34 was used as marker for hematopoietic stem cells [21]. We found that CD44, CD73, CD90 and CD105 were all positively expressed in hMSCs while CD34 was negatively expressed, which implies the successful isolation and culture of hMSCs (Figure 6A). The ability of hMSCs to induce differentiation was then evaluated *in vitro*. After a cell culture period of 2 weeks, we found the presence of a large number of lipid droplets inside the cells. The Oil red O staining confirmed that hMSCs had the function of adipogenic differentiation. The Alizarin red S staining showed that after 4 weeks of osteogenic differentiation, the cell structure became unclear, along with a large number of red calcareous deposits (Figure 6B–6C).

The presence of exosomes was isolated from the supernatant of hMSCs, and confirmed under a transmission electron microscope (TEM). The findings revealed the existence of exosomes in the supernatant of hMSCs. The shape of exosomes was solid and compact, and the majority was either cup-shaped or appeared globular. The sizes of the exosomes ranged from 50 to 200 nm (Figure 6D). Western blot analysis was employed for detection of CD63 protein which is a marker present on the surface of exosomes. We found that the expression of CD63 in exosomes was significantly higher than that in hMSCs which was used as the negative control (NC). These results further confirmed the success of exosome extraction (*p* < 0.05) (Figure 6E).

### MSCs deliver miR-199a to glioma cells by secreting exosomes

**Figure 7:**
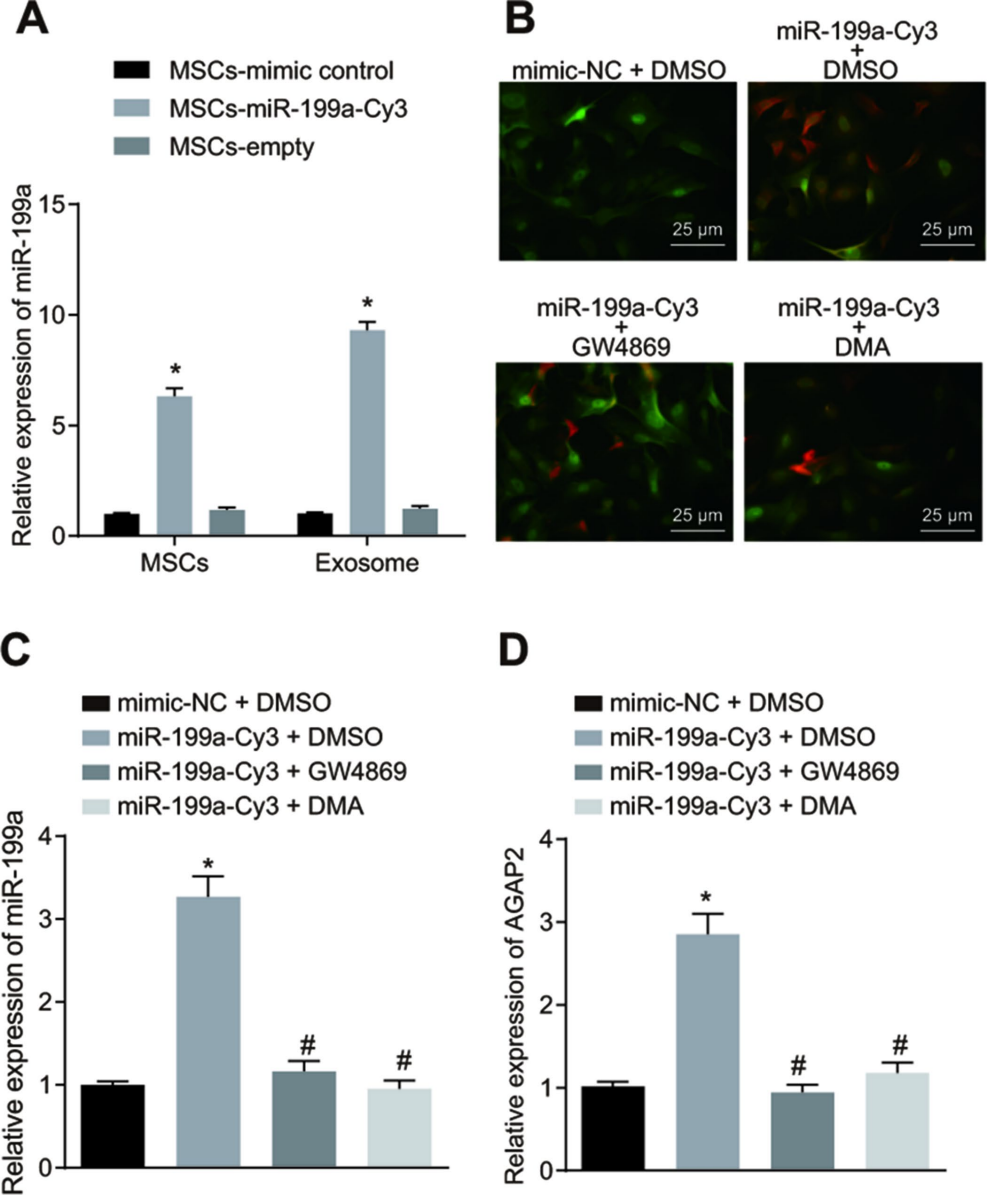
**The hMSCs deliver miR-199a to glioma cells by secreting exosomes.** (**A**) the expression of miR-199a in the presence of MSC-miR-199a, MSC-miR-control and MSC-empty, determined by RT-qPCR. (**B**) miR-199a could be delivered to U251 glioma cells form hMSCs, verified by fluorescence microscopy detection (scar bar = 25 μM). (**C**) the expression of miR-199a in U251 cells after co-culture, determined by RT-qPCR. (**D**) the expression of AGAP in U251 cells after co-culture, determined by RT-qPCR. * *p* < 0.05 compared with the MSCs + mimic control group or the mimic-NC + DMSO group; # *p* < 0.05 compared with the miR-199a-Cy3 + DMSO group. The results were measurement data, presented as mean ± standard deviation. One-way analysis of variance was used for comparison among multiple groups. The experiment was repeated three times. RT-qPCR, reverse transcription quantitative polymerase chain reaction; MSCs, mesenchymal stem cells. mimic-NC + DMSO, cells treated with mimic-negative control and dimethyl sulphoxide; miR-199a-Cy3 + DMSO, cells treated with miR-199a-Cy3 and dimethyl sulphoxide; miR-199a-Cy3 + GW4869, cells treated with miR-199a-Cy3 and GW4869; miR-199a-Cy3 + DMA, cells treated with miR-199a-Cy3 and DMA.

U-251 cells were transfected with pCDNA3.1-GFP, and hMSCs were transfected with Cy3-labelled miR-199a mimic (miR-199a-Cy3). The expression of miR-199a was measured in the MSC-miR-199a, MSC-NC and MSC-blank groups by RT-qPCR. We found a significant increase in miR-199a expression in hMSCs transfected with miR-199a-Cy3 compared to those transfected with miR-199a-NC and without any transfection (Figure 7A). This suggests that hMSCs can effectively release exosomes containing miR-199a. In order to prove that hMSCs deliver miR-199a to glioma cells by secreting exosomes, the hMSCs presented with Cy3 that were labeled as over-expressed miR-199a and U251 cells exhibited green fluorescence were co-cultured and observed under the fluorescence microscopy. The combined images showed that the transfected hMSCs could effectively transfer miR-199a into U251 cells, thereby highlighting the presence of exosomes (Figure 7B). Next, we employed GW4869 and DMA to reduce the exosome secretion for determination of the function of exosomes in this process. As illustrated in Figure 7C, the miR-199a expression was significantly inhibited in U251 cells during the co-culture experiment with GW4869 or DMA. To determine whether delivered miR-199a *in vitro* could effectively inhibit endogenous AGAP2 in glioma cells, the AGAP2 level in glioma cells co-cultured with hMSCs containing Cy3-labelled miR-199a was measured by RT-qPCR. AGAP2 expression in U251 cells was effectively inhibited by miR-199a *in vitro*, and this effect was significantly hindered by GW4869 and DMA (Figure 7D). Our results from this part of the study thereby demonstrate that exosomes contribute to miR-199a delivery from HMSCs into glioma cells via exosomes.

### The proliferation, invasion and migration of glioma cells was inhibited by MSCs-delivered miR-199a *via* exosomes

**Figure 8:**
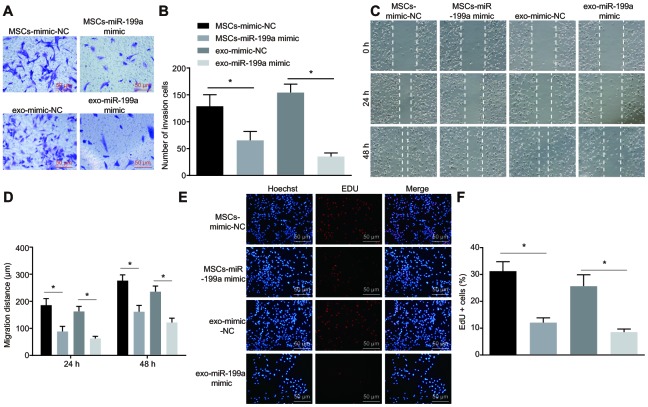
**MSCs-derived exosomal miR-199a inhibits the proliferation, invasion and migration of glioma cells.** (**A**–**B**) the invasion ability of U251 cells, assessed by Transwell assay (Scale bar = 50 μM). (**C**–**D**) the migration ability of U251 cells, measured by scratch test (Scale bar = 100 μM). (**E**–**F**) the proliferation ability of U251 cells, evaluated by EdU assay (Scale bar = 50 μM). * *p* < 0.05. The results were measurement data, presented as mean ± standard deviation. Paired *t*-test was used for the analysis. The experiment was repeated three times. MSCs-mimic-NC, glioma cells co-cultured with mesenchymal stem cells and transfected with mimic-negative control; MSCs-miR-199a mimic, glioma cells co-cultured with mesenchymal stem cells and transfected with miR-199a mimic.

To determine whether the miR-199a released from the exosomes of hMSCs could regulate the biological function of U251 cells, the proliferation, invasion and migration abilities of U251 cells co-cultured with hMSCs or with hMSCs-derived exosome were analyzed by EdU assay, Transwell assay and scratch test. The results showed that the proliferation, invasion and migration of U251 glioma cells were inhibited after miR-199a treatment in a co-culture system with either hMSCs or hMSCs-derived exosomes (Figure 8A–8F). This suggests that hMSCs could inhibit the proliferation, invasion and migration of glioma cells by delivering miR-199a to U251 cells, thereby exerting an anti-tumor effect *via* release of exosomes.

### Transfer of miR-199a from hMSCs enhances the chemosensitivity to glioma

**Figure 9:**
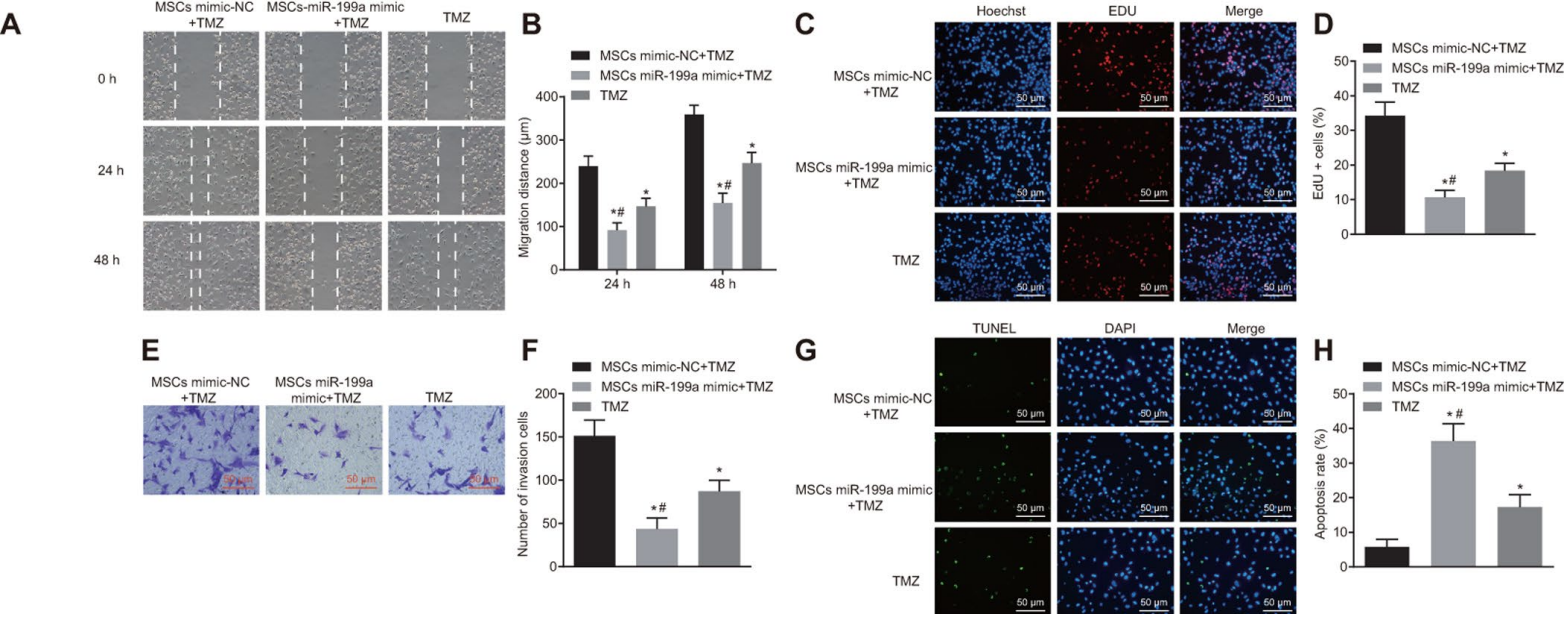
**Combination of miR-199a and hMSCs delivering miR-199a enhances the chemosensitivity of glioma cells to TMZ.** (**A**–**B**) the migration ability of U251 cells, assessed by scratch test (Scale bar = 100 μM). (**C**–**D**) the invasive ability of U251 cells, measured by Transwell assay (Scale bar = 50 μM). (**E**–**F**) the proliferation ability of U251 cells, evaluated by EdU assay. (**G**–**H**) the glioma cell apoptosis, detected by TUNEL staining (Scale bar = 50 μM). * *p* < 0.05 compared with the mimic-NC + TMZ group; # *p* < 0.05 compared with the TMZ group. The results were measurement data, presented as mean ± standard deviation. Paired *t*-test was used for the analysis. The experiment was repeated three times. TMZ, cells treated with Temozolomide; mimic-NC + TMZ, cells treated with mimic-negative control and Temozolomide; miR-199a mimic + TMZ, cells treated with miR-199a mimic and Temozolomide.

To investigate the chemotherapeutic effect of over-expressed miR-199a on the glioma, U251 cells were treated with MSCs-miR-199a mimic and temozolomide (TMZ) *in vitro*. Migration and invasion of U251 cells were assessed by scratch test and Transwell assay. We found that TMZ alone could inhibit the migration and invasion of glioma cells, and that the addition of MSCs-miR-199a helped strengthened the inhibition potency (Figure 9A–9D). Meanwhile, the viability of U251 cells was evaluated after the treatment of TMZ by EdU assay. Results showed that TMZ alone could significantly inhibit the viability of U251 cells, while miR-199a delivered by hMSCs enhanced the inhibitory effects (Figure 9E–9F). To investigate the effects of the combination of TMZ and hMSCs on glioma cell apoptosis, terminal deoxynucleotidyl transferase-mediated dUTP nick-end labeling (TUNEL) analysis was conducted. The results revealed that hMSCs delivery of miR-199a, when combined with TMZ, significantly promoted U251 cell apoptosis compared to the single treatment with hMSCs miR-NC or TMZ (Figure 9G–9H). Overall, delivering of miR-199a by hMSCs enhanced the chemosensitivity to glioma.

### Combination of miR-199a and hMSCs inhibits tumor growth *in vivo*

**Figure 10:**
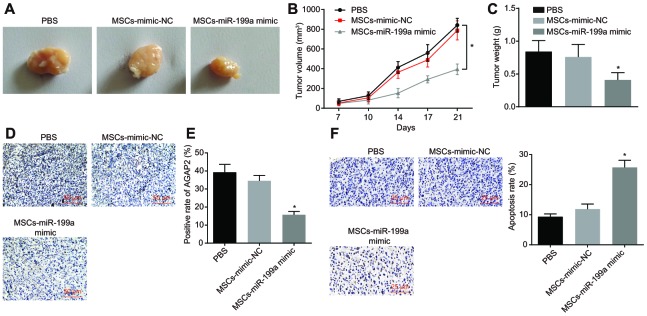
**Combination of miR-199a treatment and hMSCs containing miR-199a inhibits tumor growth *in vivo*.** (**A**) the tumor sizes in tumor-bearing nude mice after treatment with PBS, MSCs-mimic NC, MSCs-miR-199a mimic. (**B**) the tumor volume in tumor-bearing nude mice after treatment with PBS, MSCs-mimic NC, MSCs-miR-199a mimic. (**C**) the tumor weight in tumor-bearing nude mice after treatment with PBS, MSCs-mimic NC, MSCs-miR-199a mimic. (**D**–**E**) the changes of AGAP2 expression, detected by immunohistochemistry (Scale bar = 50 μM). (**F**) the apoptosis of glioma cells in tumor-bearing nude mice, detected by TUNEL staining (Scale bar = 25 μM). * *p* < 0.05 compared with the PBS group. The results were measurement data, presented as mean ± standard deviation. Repeated measures of variance of analysis was used for the analysis in B and one-way variance of analysis was used for the other analyses. The experiment was repeated three times. PBS, cells treated with phosphate buffered saline; MSCs-mimic NC, mesenchymal stem cells transfected with mimic negative control; MSCs-miR-199a mimic, mesenchymal stem cells transfected with miR-199a mimic.

To further evaluate the carrier effect of hMSCs and whether miR-199a still had obvious anti-tumor effect *in vivo*, MSCs transfected with miR-199a mimic (MSCs-miR-199a mimic), MSCs transfected with mimic NC (MSCs-mimic-NC) or phosphate buffered saline (PBS) was subcutaneously injected into tumor-bearing nude mice. The changes of tumor volume and weight were measured in nude mice, and immunohistochemistry was performed on the tumor tissues of nude mice. The results highlighted that the volume and weight of tumors in mice injected with MSCs-miR-199a mimic were distinctly smaller than those in mice injected with MSCs-mimic-NC or PBS (Figure 10A–10C). This demonstrates the inhibitory function of miR-199a on tumors *in vivo*. In addition, immunohistochemistry analysis revealed AGAP2 expression was significantly lower in the mice injected with MSCs-miR-199a mimic relative to other experimental groups (Figure 10D, 10E). These results further confirmed that miR-199a could be transferred from hMSCs to glioma cells, thereby reducing the expression of AGAP2 in glioma tissues. The effect of overexpressed miR-199a on the apoptotic rate of U251 cells was assessed by TUNEL assay and showed an increase in the apoptotic rate of U251 cells after treatment with MSCs-miR-199a mimic compared to those treated with either MSCs-mimic-NC or PBS (Figure 10F). It was clarified that the up-regulation of miR-199a promoted the apoptosis of glioma cells *in vivo*.

## DISCUSSION

Gliomas are commonly occurring primary brain tumors that remain rarely curable [22]. Exosomes derived from MSCs transfected with miRNAs have been found to be promising therapeutic tools for glioma therapy [23]. In this study, we explored the role of MSCs-derived exosomes delivering miR-199a in gliomas. The results demonstrate that MSCs-derived exosomes could deliver miR-199a to glioma cells to inhibit the progression of glioma by regulating AGAP2.

Initially, we found that miR-199a was expressed at low levels, while AGAP2 was highly expressed in glioma. We also found that AGAP2 was the target gene of miR-199a when predicted by the bioinformatics website and verified by the dual luciferase reporter gene assay. Consistent with our results, the expression of miR-199a was also found to be down-regulated in central nervous system primary lymphomas [24]. Moreover, a lower expression of miR-199a-3p was also determined in glioma samples compared to that in normal brain tissues [25]. Another study also showed that miR-199a-3p expression was found to be in lower amounts in glioma tissues relative to that in the adjacent non-tumor tissues [26]. Consistently, AGAP2 was identified as a putative oncogene for glioblastoma multiforme [27]. Moreover, another GTPase, known as ADP-ribosylation factor 6, also showed its high expression in invasive human glioma cells [28].

In our study, we also found that over-expression of miR-199a or AGAP2 silencing was led to inhibition of cell invasion, migration as well as proliferation of glioma cells. As previously reported, miR-199a-3p is capable of suppressing the proliferation of glioma cells through the regulation of the AKT/mTOR signaling pathway [25]. Moreover, miR-199 is also able to suppress the cell proliferation, migration and invasion in hepatocellular carcinoma via the negative regulation of RGS17 [29]. Arf GAPs are established as nonredundant mediators of specialized membrane surfaces that take place during cell migration [30]. Knowing this, it is not surprising to postulate that Arf GAPs have the potential of promoting progression and migration of malignant cancer cells. For instance, ARFGAP3 is detected to be able to facilitate the proliferation and migration of prostate cancer cells [31]. In this study, we elucidated that miR-199a inhibited the progression of glioma cells *via* down-regulation of AGAP2.

In the latter part of our study, we demonstrated that hMSCs were able to deliver miR-199a to the glioma cells through exosomes, which inhibited glioma cell proliferation, invasion and migration. MSCs have been discovered to be capable of delivering synthetic miRNA mimics to glioma cells and glioma stem cells that result in the suppression of migration and self-renewal abilities of these malignancies [32]. Many types of miRNAs have been reported to be loaded by exosomes and play key roles in all types of cancers. For instance, MSCs can be used in glioma treatment, by serving as natural bio-factories for exosomes that carry miR-124a [33]. In line with our findings, exosomes derived from MSCs expressing miR-146b inhibit also glioma growth [34]. MSC-derived exosomes containing miR-584 are identified to be conducive for cancer therapy [23]. In addition, MSCs-derived exosomes are capable of suppressing the development of hepatocellular carcinoma [35]. Furthermore, our study showed that the therapy of miR-199a-loaded MSCs enhanced the chemosensitivity to glioma and inhibited the tumor growth *in vivo*. Consistently, exosomes derived from MSCs that are modified by miR-122 are able to enhance the chemosensitivity of hepatocellular carcinoma as previously reported [36]. The combination of both miR-200c with IL-21-secreting human umbilical cord MSCs exerts an inhibitory role not only in the tumor growth and metastasis but also in the epithelial-mesenchymal transition (EMT) in epithelial ovarian cancer [37].

**Figure 11:**
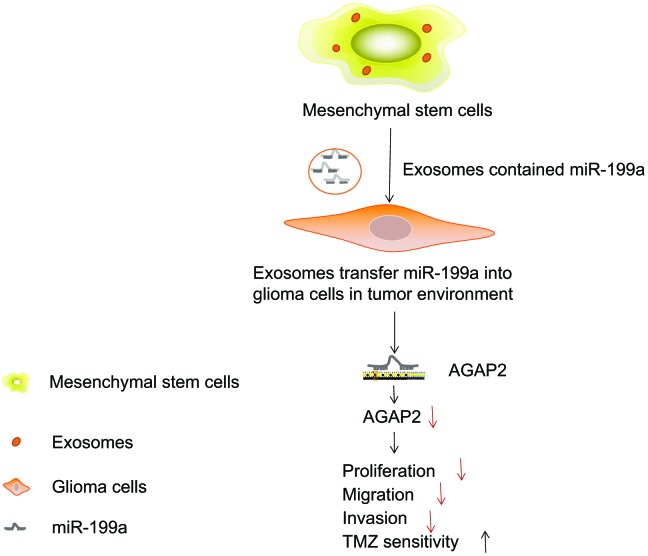
**MSCs-derived exosomes deliver miR-199a to glioma cells inhibiting the proliferation, migration and invasion of glioma cells, as well as enhancing the chemosensitivity by down-regulating AGAP2.**

In summary, this study demonstrates that miR-199a, when delivered by hMSCs *via* the exosomes, is able to negatively regulate the expression of AGAP2, thereby inhibiting the proliferation and enhancing apoptosis of glioma cells (Figure 11). These findings are likely to provide a novel molecular target for glioma treatment. However, the specific mechanism by which AGAP2 exerts an influence on glioma cells still lacks clarification. The efficient delivery of the miR-199a from the MSCs to glioma cells *via* the exosomes still lack supporting data. Therefore, further studies are required to validate and substantiate our findings from this study.

## MATERIALS AND METHODS

### Ethics statement

This study was conducted with the approval of the Ethics Committee of Nanfang Hospital, Southern Medical University. All patients have signed written informed consents prior to enrollment. Animal experiment procedures were performed in strict accordance with the protocols approved by the Animal Ethics Committee.

### In silico prediction

We used the GEO database (https://www.ncbi.nlm.nih.gov/geo/) to retrieve glioma-related expression chips, and used R-language "limma" package for differential genes analysis. The parameters of |logFC| > 2 and p value < 0.05 were used as the screening criteria for differential genes. The regulatory miRNAs targeting GAP2 was predicted by the TargetScan database (http://www.targetscan.org/vert_71/), the mirDIP database (http://ophid.utoronto.ca/mirDIP/index.jsp#r) and the StarBase database (http://ophid.utoronto.ca/mirDIP/index.jsp#r).

### Tissue sample collection

There were 92 normal brain tissues and glioma tissue specimens (17 normal brain tissues and 75 glioma tissues) collected from patients who underwent surgical resection at the Neurosurgery Department of Nanfang Hospital, Southern Medical University from 2015 to 2018. Among these glioma cases, there were 31 females and 44 males. Based on the glioma grading criteria published by WHO in 2016 [38], 27 glioma specimens belonged to grade II (21 cases of astrocytoma, 6 cases of oligodendroglioma), 25 glioma specimens in grade III (21 cases of anaplastic astrocytoma, 4 cases of anaplastic ependymoma), and 23 specimens in grade IV (23 cases of glioblastoma). The average age of patients with low-grade glioma (grade II) was 44.70 ± 9.09 years at the time of operation, and that of patients with high-grade glioma (grade III, IV) was 46.52 ± 10.19 years. Patients that met the following criteria were included in the study: diagnosis of glioma was confirmed through pathological examination post operation; no cancer treatment was undertaken before operation; complete resection of all tumor nodules was confirmed by the absence of glioma tissues on the surface through pathological examination; complete patient clinicopathological data and follow-up data were provided. Patients were excluded from this study if they died from non-glioma related diseases or other forms of accidents.

### Cell culture

Normal human astrocytes (NHAs) and six glioma cell lines (U251, LN229, T98G, LN-18, SF-539 and A172) were purchased from the cell bank of BeNa Culture Collection (BNCC; Suzhou, China) available at http://www.bncc.org.cn/plist/p1-0-0-1.html. Human glioma cell lines were incubated in Dulbecco's modified Eagle medium (DMEM) containing 10% fetal bovine serum (FBS) in an incubator at 37°C, with 95% relative humidity, 5% CO_2_ and 95% O_2_. The cells were regularly observed under a light microscope and the culture medium was replaced according to the growth state and confluency of cells. Cells were passaged when cell confluence reached approximately 70% to 80%. After removing culture medium, the cells were rinsed twice with PBS and detached with 1 to 2 mL 0.25% trypsin for 20 to 30 seconds. Next, 2 mL cell culture medium containing 10% FBS was added to immediately stop digestion, and the culture dish was shaken gently to suspend all the cells. Following centrifugation at 200 ×g for 5 minutes, the cells were rinsed twice with PBS and the supernatant was subsequently discarded. Culture medium was re-added to the cells in order to make create a single cell suspension and then seeded into a new culture flask. The cells were incubated in the incubator for further use.

### Isolation and identification of hMSCs

At first, 10 mL bone marrow was extracted from the femoral shaft fracture end using a 20 mL syringe (containing 2000 IU heparin) under sterile conditions and quickly mixed with heparin. The bone marrow was centrifuged at 300 ×g for 10 minutes and the supernatant containing the upper adipose tissues was removed followed by rinsing with DMEM thrice. Finally, 15 mL medium was then used to resuspend the cells, and the same volume of the Ficoll-Paque^TM^ Plus lymphocyte separation solution (density: 1.077 g/mL) was slowly added to the centrifuge tube. This was proceeded by centrifugation for 20 minutes at 900 ×g. The nucleated cells were located in the interface and the upper layer of liquid; most of the red blood cells were deposited at the bottom. Nucleated cells were obtained from the interface using a stalk, washed three times with 30 mL PBS, centrifuged for 8 minutes at 300 ×g and washed twice, removing the supernatant afterwards. Next, 5 mL cell culture medium was then added to uniformly diffuse the cells. After that, 10 μL cell suspension was added to 490 μL PBS, and evenly mixed. Next, 10 μL cells were extracted and then counted under a light microscope. Cells were then seeded in a culture flask (1 × 10^5^ per flask) and incubated with 5 mL low-sugar medium at 37°C, with 5% CO_2_ and saturated humidity. The positive markers CD44 (ab6124, Abcam Inc., Cambridge, UK), CD73 (ab202122, Abcam Inc., Cambridge, UK), CD90 (ab23894, Abcam Inc., Cambridge, UK) and CD105 (ab114052, Abcam Inc., Cambridge, UK) and the negative marker CD34 (ab8536, Abcam Inc., Cambridge, UK) of hMSCs were detected using flow cytometry.

### Identification of adipogenic and osteogenic differentiation *in vitro*

The cells at passage 2 in logarithmic growth phase were collected and cultured in 6-well plates (1 × 10^5^ cells/mL). After the cell confluence reached 70%, the culture medium in the adipogenic induction group was replaced with the adipogenic induction medium (containing 1 μM rosiglitazone, 1 μM dexamethasone, 0.5 mM 3-isobutyl-1-methyl-xanthine, 10 μg/mL insulin, 0.2 mM indomethacin, 1% penicillin/streptomycin plus glutamine and 10% FBS (Cyagen Biosciences, Guangzhou, China). The complete culture medium was used for cell culture in the blank control, replacing the medium every 3 days. The proliferation and differentiation of the cells were observed with an inverted phase contrast microscope (Olympus, Tokyo, Japan). The induction of adipogenic differentiation of hMSCs was followed by the removal of culture medium as well as two washes with PBS. Subsequently, 2 mL of 4% neutral formaldehyde was added into each well and was left to fix for 30 minutes at 4°C. After discarding the formaldehyde, hMSCs were rinsed twice with PBS and dyed using 1 mL oil red O (Amresco, Solon, OH, USA) for 30 minutes. The oil red O was discarded, and the hMSCs subsequently rinsed twice with PBS. Lipid droplet formation was observed under an inverted phase-contrast microscope.

The cells at passage 2 in logarithmic growth phase were selected and seeded into 6-well plates with a cell density of 1 ×10^5^ cells/mL. When the cell confluence reached 70%, the medium in each well was replaced with the osteogenic induction medium [containing 0.17 mM-L ascorbic acid; 5% FBS, 10 mm beta-glycerol phosphoric acid, 100 nM dexamethasone (Sigma-Aldrich Chemical Company, St Louis, MO, USA), 1% penicillin/streptomycin (Cyagen Biosciences, Guangzhou, China)]. The cells were cultured with the complete culture medium in the blank control which was replaced every 3 days. Cell proliferation and differentiation were observed and recorded every day using an inverted phase-contrast microscope. On the 21^st^ day after osteogenic induction, Alizarin red S (Amresco, Solon, OH, USA) staining was carried out. The cells were washed with Dulbecco’s phosphate buffered saline (DPBS; Gibco, Carlsbad, California, USA) twice and fixed with 4% paraformaldehyde for 10 minutes. After removing paraformaldehyde, the cells were rinsed twice with DPBS. A suitable amount of 0.1% Alizarin red S solution was added to the cell slides. After incubation for 30 minutes at 37°C, the Alizarin red S solution was removed, followed by 2 washes with DPBS. Finally, the cell slides were dried out using the filter paper, and the staining of calcium nodules was observed and photographed under an optical microscope.

### Plasmid transfection

The day before transfection, glioma cells were inoculated into a 6-well plate (2 × 10^5^ cells/well), 60-mm plate (5 × 10^5^ cells/plate) or 100-mm plate (2 × 10^6^ cells/well). Transfection was conducted when the cell confluence reached 60% to 80%. Next, miR-199a mimic, miR-199a inhibitor, shRNA targeting AGAP2 (shAGAP2), AGAP2 over-expression plasmid and their negative controls including miR-NC, inhibitor-NC and shNC (GenePharma, Shanghai, China) were transfected into glioma cells respectively. The transfection protocol was performed according to the instructions of the Lipofectamine 2000 kit (Invitrogen, Carlsbad, CA, USA).

### Dual luciferase reporter gene assay

The targeting relationship between miR-199a and AGAP2 was verified by a dual luciferase reporter gene assay. Glioma cells were inoculated into a 24-well plate and then transfected with WT plasmid and MUT plasmid a day before transfection. The 3’-UTR sequences of the WT AGAP2 and the MUT AGAP2 were synthesized and integrated into psiCHECK-2 dual-luciferase vector to construct psiCHECK-2-AGAP2-3’-UTR-WT (AGAP2-WT) and psiCHECK-2-AGAP2-3’-UTR-MUT (AGAP2-MUT), respectively. Based on the manufacturer's instructions, the cells were transfected using Lipofectamine 2000 (Invitrogen, Carlsbad, CA, USA). The 800 ng psiCHECK-2-AGAP2-3’-UTR vector was co-transfected with 50 pmol miR-199a mimic, miR-199a inhibitor, inhibitor-NC or miR-NC. The luciferase activity was detected using a dual luciferase reporter kit (Promega, Madison, WI, USA) 24 hours after transfection. All investigations involving at least 6 wells were repeated in triplicates.

### Isolation and characterization of exosomes

The culture medium of hMSCs was collected and centrifuged at 300 ×g for 5 minutes, at 2000 ×g for 10 minutes, and at 10000 ×g for 35 minutes. The supernatant was collected and filtered by a 0.22 μm filter film (Merk Millipore, Billerica, MA, USA) and then ultra-centrifuged at 100000 ×g for 2 hours. The collected sediments were washed with 20 mL cold PBS, and then purified by centrifugation at 100000 ×g for another 2 hours. All ultracentrifugation steps were conducted at 4°C in a Beckman ultracentrifuge (Optima L-90K; Beckman Instruments, Fullerton, CA) with SW-32Ti rotor. The temperature during the whole ultracentrifugation process was controlled at 4°C. Finally, the spherical samples were resuspended in 50 to 100 μL PBS and stored at -80°C. To carry out the electron microscopy analysis, the isolated samples were cultured in PBS [21]. The samples were first adsorbed onto a carbon coated nickel grid and counterstained with 2% tungstate for 5 minutes. Next, the stains were removed from the grid with fire paper and the samples were washed twice with distilled water. Samples were then examined under a JEM-1230 electron microscope (Nihon Denshi, Tokyo, Japan) after drying (the acceleration voltage was 80 thousand volts).

Specific inhibitors GW4869 (Sigma-Aldrich Chemical Company, St Louis, MO, USA) and DMA (Santa Cruz Biotechnology, Inc, Santa Cruz, CA, USA) were employed to block exosome secretion. To confirm that the miRNA was delivered via exosomes, the cells were first treated with 10 nM GW4869 or 15 nM DMA, with dimethyl sulphoxide (DMSO) as the NC. MSCs transfected with miR-199a were placed in a 6-well plate. The cells treated with GW4869, DMA or DMSO were co-cultured with the transfected glioma cells in a 6-well plate. After 48 hours of culture, the culture medium was collected for isolation of exosomes.

### Co-culture of hMSCs or exosomes and glioma cells

Glioma cell line U251 was transfected with fluorescent labeled pCDNA3.1-GFP. hMSCs were transduced with Cy3-labeled miR-199a (miR-199a-Cy3) (GenePharma, Shanghai, China). After 12 hours of transfection, hMSCs and U251 were mixed at a ratio of 1 : 1 and seeded into the 96-well plate (100 cells/well) for 2 days. Flow cytometry was applied to sort the cells. The results were photographed and observed under a fluorescence microscope. Finally, 20 μg exosomes were co-cultured with glioma cell line U251 for 48 hours.

### RT-qPCR

The total RNA was extracted from the cells and tissues by a TRIzol reagent (Invitrogen, Carlsbad, CA, USA). The All-in-One miRNA RT-qPCR kit (GeneCopoeia, Rockville, MD, USA) was used to conduct RT-qPCR analysis. In accordance with the manufacturer’s instructions (LightCycler 480, Roche Diagnostics GmbH, Mannheim, Germany), the RNA was reversely transcribed into complementary DNA (cDNA). The primers shown in Table 1 were synthesized by Takara Bio Inc. (Otsu, Shiga, Japan). U6 was used as the internal reference for miR-199a (miR-199a-5p primer ID, hsmq-0715, Catalog#HMIQP 0289; U6 primer ID, hsRNAU 6, Catalog#HMIQP 9001; LightCycler 480, Roche Diagnostics GmbH, Mannheim, Germany); glyceraldehyde-3-phosphate dehydrogenase (GAPDH) was used as the internal reference for AGAP2. The relative expression of miR-199a and AGAP2 was determined by the SYBR Green real-time RT-qPCR method and normalized. The fold changes were calculated by means of relative quantification (2^-△△Ct^ method).

**Table 1:** Primer sequences for RT-qPCR.

**Genes**	**Primer sequences (5'-3')**
miR-199a	F: 5'-ACACTCCAGCTGGGTCCCTGAGACCCTTTA-3'
	R :5'-CTCAACTGGTGTCGTGGAGTCGGCAATTCAGTTGAG-3'
AGAP2	F: 5'-GCAGCTACTATGAGACTTGTGC-3'
	R: 5'-GTGACCAACATTCGGTGAGGA-3'
U6	F: 5'-CGCTTCGGCAGCACATATACTA-3'
	R: 5'-CGCTTCACGAATTTGCGTGTCA-3'
GAPDH	F: 5'-AGAAGGCTGGGGCTCATTTG-3'
	R: 5'-AGGGGCCATCCACAGTCTTC-3'

Note: RT-qPCR, reverse transcription quantitative polymerase chain reaction; F, forward; R, reverse; miR-199a, microRNA-199a; AGAP2, Arf GTPase-activating protein-2; GAPDH, glyceraldehyde-3-phosphate dehydrogenase.

### Western blot analysis

Western blot analysis was conducted in accordance to protocols from a previous report [39]. The total proteins in cells and tissues were extracted using the radio-immunoprecipitation assay (RIPA) cell lysis buffer (R0010; Beijing Solarbio Science & Technology Co., Ltd., Beijing, China) containing phenylmethylsulfonyl fluoride (PMSF). The concentration of protein was measured by a bicinchoninic acid (BCA) protein analysis kit. The protein samples were separated by 6% to 15% sodium dodecylsulfate (SDS)-polyacrylamide gel electrophoresis (PAGE). The separated proteins were then transferred onto the polyvinylidene fluoride (PVDF) membrane. The membrane was then blocked with 5% bovine serum albumin (BSA; AMRESCO, Solon, OH, USA) for 1 hour. Primary rabbit antibodies to AGAP2 (ab224118, 1 : 1000, Abcam Inc., Cambridge, MA, USA) and CD63 (ab134045, 1 : 1000, Abcam Inc., Cambridge, MA, USA) were added to the membrane, and incubated at 4°C overnight. On the following day, the membrane was washed with Tris-buffered saline with Tween 20 (TBST) three times. After that, goat anti-rabbit immunoglobulin G (IgG) antibody (1 : 1000; A21020, Abbkine, CA, USA) was added onto the membrane and incubated at 37°C for 1 hour. All the primary antibodies were purchased from Abcam company (Abcam Inc., Cambridge, MA, USA). The membrane was washed by TBST 3 times at room temperature, 5 minutes each time, followed by development with enhanced chemiluminescence (ECL) reagent. The ratio of the gray value of the target band to GAPDH was representative of the relative protein expression and analyzed with an image analyzer.

### Scratch test

The U251 cells (8 × 10^4^) were seeded into the 6-well plate and cultured for 24 hours. MiR-199a and miR-NC were then transfected into cells using Lipofectamine 2000 (Invitrogen, Carlsbad, CA, USA). After 48 hours, when the cell confluence reached 90%, a scratch was made on the top of the center of the converging cells using a pipette tip. The cell migration distance at 24 and 48 hours after scratching was measured and recorded under a microscope.

### Transwell assay

Matrigel (YB356234, Shanghai Yu Bo, Shanghai, China) that was preserved at -80°C was extracted and balanced at 4°C overnight to melt into liquid. Then, 200 μL Matrigel was added to 200 μL serum-free medium, and evenly mixed to dilute the Matrigel. Next, 50 μL Matrigel was added into the apical chamber of each Transwell plate and left to incubate for 2 to 3 hours until the Matrigel solidified. The cells were then detached and counted, and a cell suspension was created using 20% FBS medium. Thereafter, 100 μL cell suspension was added into the apical chamber of each well and 800 μL medium containing 20% FBS was added into the basolateral chamber. After incubation at 37°C for 20 to 24 hours, the Transwell plate was taken out, rinsed twice with PBS, washed with formaldehyde for 10 minutes and rinsed with water 3 times. The Transwell plate was then stained with 0.1% crystal violet and allowed to sit at room temperature for 30 minutes, followed by rinsing with PBS twice. The cells on the inner surface were wiped off with cotton balls. Cells were subsequently observed, photographed and counted under an inverted light microscope.

### EdU assay

The EdU incorporation test was performed using a Cell-Light EdU imaging test kit (RiboBio, Guangzhou, China). After treatment for 24 hours, the U251 cells were sorted from the co-culture system, washed twice with PBS, and inoculated into a 96-well plate (5 × 10^3^ cells/well). After 6 hours, the medium containing EdU was added to the plate and incubated for 60 minutes. Next, the cells were fixed with 4% formaldehyde for 15 minutes, treated with 0.5% Triton X-100 for 20 minutes, and stained with Apollo staining solution for 30 minutes. In each well, the cell DNA was stained by 5 μp/mL Hoechst 33342 up to 30 minutes. Images were obtained and analyzed under a fluorescence microscope. The excitation fluorescence of Hoechst 33342 was analyzed at 350 nm and the emission fluorescence was measured at 461 nm.

### Apoptosis detection by TUNEL assay

The apoptotic cells were detected by TUNEL staining with the *in situ* cell apoptosis detection kit (Takara Bio Inc., Otsu, Shiga, Japan). U251 cells were treated with adriamycin and incubated either under hypoxic or normoxic conditions. After the treatment, the culture medium and adherent cells were collected. The suspended cells were collected after centrifugation, and the adherent cells were detached by trypsin and transferred into the same tube containing the suspended cells. Cell pellets were then collected through centrifugation. Ten regions were randomly selected to count the number of the positive cells.

### TMZ treatment

To determine the effect of miR-199a on the resistance of U251 cells to TMZ, U251 cells underwent overnight incubation in the 96-well plate at 37°C. After 24 hours, U251 cells were transfected with miR-199a mimic and treated with 100 μM TMZ. The apoptosis of U251 cells was tested by the TUNEL assay, the proliferation pattern was tested by the EdU test, and migration and invasion abilities were evaluated by scratch test and Transwell assay.

### Tumor xenograft in nude mice

Female Balb/c nude mice (4 - 6 weeks, 15 - 20 g), purchased from SJA Laboratory Animal Co., Ltd., Changsha, China; Certificate: SCXK (Shanghai 2012-0002), were subcutaneously injected with approximately 1 × 10^7^ U251 cells (diluted in 200 μL PBS) via the left hind limb. The volume of the tumors was measured every 3 to 4 days (twice a week). The tumor volume was calculated based on the following formula: V = A × B^2^ / 2 (mm^3^), where A represents the largest diameter and B the vertical diameter. When the tumor volume of nude mice reached approximately 100 mm^3^, the nude mice were randomly injected with PBS, hMSCs-miR-NC (hMSCs transfected with miR-NC), hMSCs-miR-199a (hMSCs transfected with miR-199a), with 6 mice in each group. After repeating the treatment process seven times, the mice were euthanized, and the tumors were excised and weighed. Excised tumors were cryopreserved in liquid nitrogen for further study or immobilized in formalin for immunohistochemistry and cell apoptosis analysis.

### Immunohistochemistry

The tumor masses of both glioma mice and clinical samples were sliced into 4-μm sections and analyzed by immunohistochemistry. The EZ Retriever system (BioCare Medical, Walnut Creek, CA, USA) was used to extract antigen for 15 minutes at 95°C - 122°C with 10 mM butyl citrate (pH 6.0). The sections were separated at 60°C, and the endogenous peroxidase activity was blocked by 0.3% H_2_O_2_ for 20 to 30 minutes in methanol. Sections were incubated at 4°C with anti-human AGAP2 antibody (Santacruz, Biotechnology, CA, USA) (dilution ratio: 1 : 250 - 1 : 500), and developed with diaminobenzidine, followed by nuclear staining with hematoxylin.

The expression of AGAP2 in tumor sections was assessed by the PER-based scoring method. The scores were calculated based on the percentage of positive nuclei present: 0 (no staining), 1 (10 - 25%), 2 (26 - 50%) or 3 (> 50%). In statistical analysis, a score of 0 was considered protein-free, and scores of 1 to 3 were considered positive for protein presence.

### Statistical analysis

All data was processed by SPSS 21.0 statistical software (SPSS, Inc., Chicago, IL, USA). Measurement data was presented as mean ± standard deviation. The comparison between glioma tissue and normal brain tissue was performed by independent sample *t*-test; the comparison between two groups was paired with *t*-test. Comparison between multiple groups was performed by one-way analysis of variance (ANOVA). The tumor volumes at varying time points were compared by repeated measures of ANOVA. *p* < 0.05 indicated that the difference was statistically significant.
